# Power management and performance optimization of underwater wireless sensor networks based on MARL

**DOI:** 10.1371/journal.pone.0343529

**Published:** 2026-03-06

**Authors:** Jingtao Guan

**Affiliations:** School of Computer Science and Engineering, Guangdong Ocean University, Yangjiang, China; Qingdao University, CHINA

## Abstract

In underwater wireless sensor network communication, communication performance degrades due to factors such as complex underwater channels and limited node resources. To reduce node redundancy energy consumption, improve transmission reliability, and extend the overall network lifetime, this study proposes an intelligent network performance optimization algorithm based on multi-agent reinforcement learning. By constructing an underwater wireless sensor network system model including fixed and mobile nodes, the network performance optimization problem is formalized as a partially observable Markov decision process. Then, multi-agent reinforcement learning is used to construct a comprehensive team reward function containing fair reuse rewards and survival time penalties, thereby establishing a distributed intelligent power management scheme. This solution enables each node to make transmission power decisions based on local observations, combined with the underlying media access control protocol, to collaboratively optimize higher-layer network performance indicators. The results show that in heterogeneous network scenarios, the proposed method achieves a network capacity of 245.68 kb and a fairness reuse index of 1.85. In imperfect networks with 5% node failures, the average communication latency is only 6.18 time slots, which is superior to the comparative algorithm. Under dynamic environments with a signal-to-interference-plus-noise ratio of 10–16 dB and a water flow velocity of 2.0 m/s, it can still maintain a network capacity of over 32,045 kb and an energy efficiency of 0.4 kb/J. These findings demonstrate that the proposed method significantly improves the robustness of underwater wireless sensor networks, providing communication support for ocean monitoring.

## 1. Background

With the increasing demand for Marine resource exploration, environmental monitoring, disaster early warning and national defense security, Underwater Wireless Sensor Networks (UWSNs), as a key infrastructure for building a “smart ocean”, are playing an irreplaceable role [1]. UWSN is composed of a large number of resource-constrained intelligent nodes deployed in complex underwater environments, capable of collaborating to collect, transmit and process high-value data. It serves as the core information network supporting critical tasks such as tsunami warning, long-term hydrological observation or seabed exploration [2,3]. Taking tsunami warning as an example, this application requires the network to achieve low-latency and highly reliable event data reporting in a highly dynamic and multi-interference underwater environment. Meanwhile, it must ensure that all monitoring nodes can work continuously and effectively over a task cycle of several months or even years [4]. This imposes almost contradictory and demanding requirements on the capacity, fairness, energy efficiency and robustness of its communication protocol. However, achieving the above-mentioned performance goals faces fundamental challenges. The propagation speed of underwater acoustic channels (~1500 m/s) is five orders of magnitude lower than that of radio waves, resulting in extremely high propagation delay (typical values range from 0.1 to 10 seconds). Meanwhile, its available bandwidth is severely limited, usually only at the kHz level [5]. These quantitative constraints determined by physical media are the fundamental challenges that the design of UWSN protocols must confront. In addition, the network itself has the basic characteristics of being distributed, strictly resource-constrained, and partially observable. Traditional methods based on static rules or optimization theories are difficult to adapt to this highly dynamic environment, often leading to unbalanced resource allocation, shortened network lifetime or sharp performance fluctuations [6]. In recent years, Reinforcement Learning (RL) has received extensive attention due to its powerful autonomous learning ability demonstrated in complex sequential decision-making problems. In particular, Multi-Agent Reinforcement Learning (MARL) is highly suitable for modeling UWSN, a typical distributed multi-user system. It enables each node (agent) to interact with the environment and other nodes, learn to make distributed decisions under partially observable conditions and collaboratively optimize global performance.

Therefore, this article proposes a distributed power control and performance optimization method for partially observable UWSNs with irrational node interference. The innovations of this work are: 1) For the first time, an accurate underwater acoustic channel model, anchor node passive movement model, and node random failure model are integrated into the MARL modeling of UWSNs, constructing a simulation environment that is more in line with physical reality. 2) A novel hybrid reward structure and semi-cooperative learning mechanism is designed. By designing a hard constrained penalty term for survival time and modeling irrational node behavior as part of environmental dynamics, the agent is guided to actively avoid interference and balance energy consumption during the learning process. 3) A centralized training distributed execution paradigm is adopted, a lightweight Value Decomposition Network (VDN) is used for training, and minimal local forward reasoning is required during the execution phase, ensuring the feasibility of deploying the algorithm on resource-constrained underwater node micro-controller (MCU).

The contribution of this study lies in: 1) Transforming the complex multi-objective optimization problem of UWSNs into a distributed power decision-making problem based on local information for each node through MARL. 2) A composite team reward function integrating fair reuse rewards and hard constraint penalties for survival time was designed, effectively guiding the agent to balance short-term throughput and long-term survival ability under local observations. By introducing a semi-cooperative learning mechanism, modeling the node failure behavior as environmental dynamics and supplementing it with local avoidance rewards, UWSNs can possess anti-interference and adaptive optimization capabilities without the need for online state communication. 3) Research has demonstrated that this upper-level intelligent power management can significantly enhance the end-to-end performance of the network layer (such as throughput, latency, and energy efficiency) by increasing the SINR at the physical layer and the transmission success rate at the link layer.

## 2. Relevant work

### 2.1. Traditional UWSN optimization methods and early learning algorithms

Early research on UWSN optimization mainly focused on designing efficient routing protocols and resource allocation strategies. For instance, the fuzzy routing protocol proposed by M. Tarif et al. [7] optimizes path selection by integrating multiple factors such as node depth and residual energy. X. Chang et al. [8] utilized the core node set to construct a virtual shortest path tree to reduce energy consumption and latency. Although such methods based on mathematical models or heuristic rules are effective in specific static scenarios, their preset parameters are difficult to adapt to the intense time-varying and spatially varying characteristics of underwater acoustic channels, and usually lack the global optimization ability for long-term network goals (such as overall lifetime).

To enhance adaptability, researchers have introduced machine learning, especially single-agent RL. For instance, C. Wang et al. [9] constructed a Q-learning framework based on link states to accelerate route convergence. Single-agent RL regards the entire network or a single decision point as a single agent and performs well on problems with a small state space. However, UWSN is essentially a distributed multi-decision entity system. Adopting a single-agent model either collects global information centrally (resulting in high communication overhead and latency) or leads to a “curse of dimensionality” due to state space explosion, making it difficult to scale to large-scale networks.

### 2.2. General framework of MARL and its application in network optimization

MARL provides a natural paradigm for optimizing distributed systems. Mainstream algorithms such as VDN [10] and Q-value Mixing Network (QMIX) [11] adopt the centralized Training Distributed execution (CTDE) paradigm. The core assumption is that the joint action value function can be decomposed into a monotonic combination of individual value functions. During the training phase, they utilize global information to learn a hybrid network to guide individual strategies. When executing, each agent relies only on local observations. Such methods have achieved great success in fully cooperative and reward-sharing games. However, its monotonicity assumption may not hold true in UWSNs, as the interference relationships between nodes can be complex and non-monotonic. Multi-agent Deep Deterministic Policy Gradient (MADDPG) [12] equips each agent with a centralized critic that can access global information during training. The critic has access to all agents’ action and state information during training, enabling it to handle more general cooperative or competitive scenarios. However, its drawback lies in that a model for estimating the policies of other agents is still required during the execution stage, and the structure of the centralized critic may become cumbersome in large-scale networks. Counterfactual Multi-Agent Policy Gradient (COMA) [13] is specifically designed for fully cooperative scenarios and performs outstandingly in tasks that require a fine assessment of individual contributions by calculating counterfactual baselines for each agent to allocate credit, thereby solving the allocation of credits among multiple agents. However, applying these advanced algorithms to UWSNs faces two core challenges: Firstly, the long latency and high loss characteristics of the underwater acoustic environment make the real-time sharing of global information (even during the training stage) costly. Secondly, most existing algorithms assume that all agents always collaborate rationally, while in real UWSNs, node failures and irrational interference are the norm.

### 2.3. Research status and limitations of MARL in UWSNs

In recent years, some studies have attempted to introduce MARL into UWSNs. For instance, to collaboratively optimize communication efficiency and reliability in resource-constrained underwater networks, T. Zhang et al. proposed a traffic load-aware resource management strategy and adopted deep MARL for its solution. The simulation results show that this strategy can adaptively achieve efficient and reliable communication under different transmission requirements and collision probability scenarios [14]. To achieve efficient multi-hop routing in overlay networks, R. A. Alliche et al. proposed the O-DQR multi-agent deep RL framework. This framework adopts a distributed training and decentralized execution mode, and enhances convergence stability through a guided reward mechanism. Experiments show that this framework is close to optimal in latency and packet loss rate, and can effectively control signaling overhead [15]. However, these works have three obvious limitations: Firstly, their algorithm designs are usually based on the ideal assumption that all nodes collaborate completely rationally, failing to effectively model and respond to irrational node behaviors in reality caused by node failures, battery depletion, or external disturbances, resulting in insufficient robustness in actual deployments. Secondly, problem modeling often ignores the dynamic characteristics of the underwater environment. For instance, it fails to fully consider the continuous impact of passive node movement caused by ocean currents on the topology and channel state, which limits the adaptability of the learning strategy. Finally, the optimization objectives are often disconnected from the core requirements of specific applications, mostly focusing on optimizing single indicators such as total throughput, and lack systematic reward designs for multi-objective collaborative optimization such as capacity and fairness under long-term survival constraints.

In conclusion, there is currently a lack of an intelligent optimization scheme for UWSNs that can simultaneously handle dynamic topologies, partially observable, and irrational node interference, and can collaboratively optimize multiple conflicting performance goals. To address the above issues, the research draws on the CTDE framework and the value decomposition ideas of VDN and QMIX, but abandons the strict monotonicity assumption to capture the complex interference relationships among underwater nodes. Compared with MADDPG, this study adopts a fully distributed execution strategy, eliminating the need to estimate or communicate strategies with other nodes during the execution phase, which is more adaptable to the high latency characteristics of underwater acoustics. Compared with COMA, this study focuses on long-term optimization of overall team rewards and satisfying hard constraints. It innovatively designs a composite reward function that integrates fair reuse rewards and survival time penalties to collaboratively optimize distributed intelligent decision-making and transmission power based on local observations of each node.

## 3. Methods

The study first establishes a system model that accurately reflects the characteristics of underwater acoustic channels and node mobility. Then, a fair reuse reward function and a centralized training distributed execution paradigm are designed to guide nodes to make collaborative decisions based on local observations, thereby jointly optimizing network capacity and communication fairness while ensuring the survival time of UWSN.

### 3.1. Construction of uwsn system model

**Fig 1:**
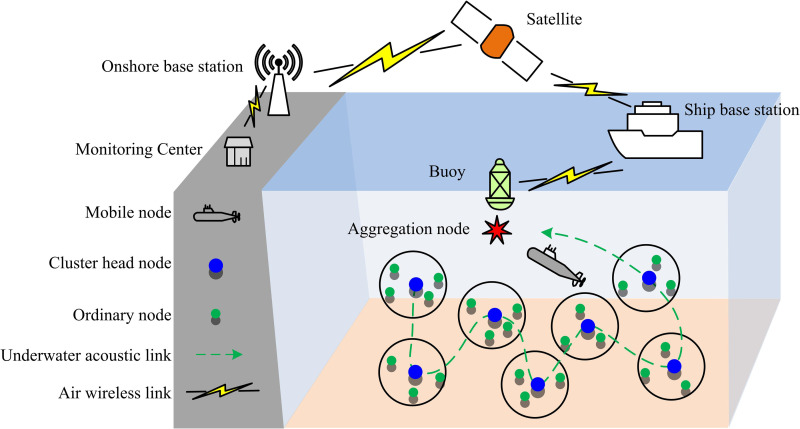
UWSN communication scenario with concurrent communication.

To achieve intelligent optimization of UWSNs, the study first establishes a system model that accurately reflects its typical characteristics and key constraints. The research focuses on four aspects: the network model, the channel model, the node mobility model, and the network constraints. Fig 1 depicts the UWSN communication scenario designed for this research.

The communication scenario shown in Fig 1 demonstrates multiple concurrent communication links existing in the network. The network contains a set of fixed sending-receiving node pairs, denoted as 𝒩={1,2,...,N}, where N=|𝒩| is the number of sending nodes in the network. Each sending node $n_{i}\in 𝒩$ is associated with a specific receiving node ${\hat{n}}_{i}$. In modeling, each sender – receiver pair ($n_{i}$, ${\hat{n}}_{i}$) can be regarded as an independent communication task. In a multi-hop network, these tasks may represent a certain hop among different multi-hop streams. For example, $n_{i}$ may be a source node and ${\hat{n}}_{i}$ is its next-hop relay node. $n_{i}$ is a relay node and is forwarding data to its next hop ${\hat{n}}_{i}$. The power optimization mechanism proposed in this study is independent of specific flow identifiers or routing paths. It acts on each active transmitting node to optimize its instantaneous transmission to a specific receiving node. Therefore, this mechanism can be seamlessly applied to the dynamic multi-hop topology established by any routing protocol, serving as a universal underlying service to enhance the reliability and efficiency of each hop transmission.

In addition, there may be mobile communication entities in the network, whose set is denoted as ℳ={1,...,M}. When these mobile nodes perform their own tasks, their communication behavior will potentially interfere with the fixed nodes. The underwater acoustic channel is one of the most complex wireless communication media known, and its characteristics directly affect the performance of UWSNs. For a sending node $n_{i}$, the signal strength at the receiving node ${\hat{n}}_{i}$ is calculated by equation(1).


$$\gamma_{i}=\frac{p_{i}h_{i,i}}{\sum_{k=1,k\neq i}^{N}p_{k}h_{k,i}+\sum_{m=1}^{M}p_{m}h_{m,i}+N\left(f\right)\Delta f}$$ (1)

In equation (1), m represents the mobile node. $\gamma_{i}$ is the Signal-To-Interference-Plus-Noise Ratio (SINR). $h_{m,i}$, $h_{k,i}$, and $h_{i,i}$ represent the channel gains of the corresponding communication links. The first two terms in the denominator are the co-channel interference from other fixed nodes and mobile nodes. N(f)Δf represents the ambient noise power, where N(f) is the noise power spectral density. $p_{m}$, $p_{k}$, and $p_{i}$ are the transmit powers of the three nodes. In this study, the monthly average temperature and salinity profile data provided by the China Argo Real-time Data Center are utilized to calculate the sound velocity profile (SSP) through the Chen-Millero sound velocity equation. Combining the Bellhop ray acoustic model, the propagation path and energy attenuation of sound waves in vertical non-uniform water bodies are simulated. Then, the channel gain h varying with time and position is obtained, which is used to reflect the temporal and spatial variability of underwater channels. The channel gain h is a function of distance d and frequency f, as shown in equation (2).


$$h=A_{\text{0}}^{-1}d^{-k}a(f{)}^{-d}$$ (2)

**Fig 2:**
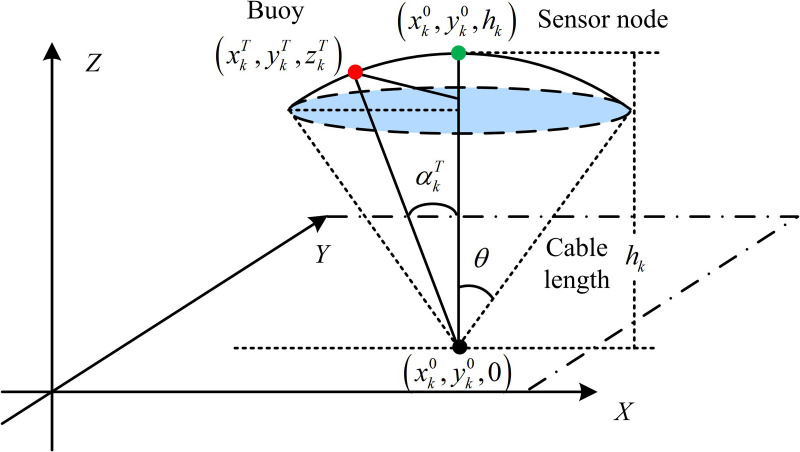
Mobility model of underwater sensor nodes.

In equation (2), $A_{\text{0}}$ is the normalization constant, k is the expansion factor (usually k=1.5), and a(f) is the absorption coefficient. This model comprehensively considers the path loss of signals and the absorption loss related to frequency, revealing the essence of high attenuation and narrow bandwidth in underwater acoustic channels. The spatiotemporal changes in temperature and salinity in the underwater environment profoundly change the propagation path and signal strength of the sound wave by affecting the Sound Speed Profile (SSP) [16]. This time-varying and space-varying sound field structure makes the channel gain h not fixed, but presents complex dynamic characteristics, further increasing the difficulty of network performance optimization [17]. In a real underwater environment, even nodes designed as “fixed” will passively move under the influence of hydrological forces such as ocean currents and tides. This movement will change the distance between nodes, thereby dynamically affecting the channel gain and network topology [18,19]. To accurately characterize this phenomenon, the study uses the tethered node model shown in Fig 2 to model the passive movement of fixed nodes.

Fig 2 simulates the random drifting behavior of a node near its anchor point, influenced by currents. The node $n_{i}$ is anchored to the seafloor $O_{i}$ by a cable of length $R_{i}$. Its velocity $v_{i}^{t}$ during a time slot t follows a normal distribution, and its direction is determined by both pitch angle $\theta_{i}^{t}$ and azimuth angle $\varphi_{i}^{t}$. Based on the current position $\left(x_{i}^{t},y_{i}^{t},z_{i}^{t}\right)$, its position $\left(x_{i}^{t+1},y_{i}^{t+1},z_{i}^{t+1}\right)$ for the next time slot is calculated using equation (3), satisfying the constraint $\|\left(x_{i}^{t+1},y_{i}^{t+1},z_{i}^{t+1}\right)-O_{i}\|\leq R_{i}$.


$$\left\{ \begin{array}{c} @l@x_{i}^{t+1}=x_{i}^{t}+v_{i}^{t}sin\theta_{i}^{t}cos\varphi_{i}^{t}\mathrm{\backslash vspace}1mm\\ y_{i}^{t+1}=y_{i}^{t}+v_{i}^{t}sin\theta_{i}^{t}sin\varphi_{i}^{t}\mathrm{\backslash vspace}1mm\\ z_{i}^{t+1}=z_{i}^{t}+v_{i}^{t}cos\theta_{i}^{t} \end{array}\right.$$ (3)

In this model, the cable length L is set to 80m to simulate the movement range of typical underwater anchor system nodes. The node velocity $v_{i}^{t}$ follows a normal distribution of $N\left(0,0.{\text{1}}^{\text{2}}\right)$ m/s, reflecting the random drift characteristics under weak ocean currents (with a velocity of approximately 0.1–0.3 m/s). The pitch angle $\theta_{i}^{t}$ and azimuth angle $\varphi_{i}^{t}$ are uniformly distributed within [−30°, 30°] to limit the swing range of the node near the anchor point. These parameters are set based on the Argo flow velocity observation data in the near-equatorial region of the Pacific Ocean and related underwater node movement studies [20,21]. The performance of UWSNs must be optimized under multiple physical and resource constraints, primarily including communication threshold constraints, energy constraints, and lifetime constraints. The communication threshold constraint ensures that data can be successfully decoded with an acceptable bit error rate. The SINR at the receiving node must be above a preset threshold $\gamma_{threshold}$, which must met $\gamma_{i}\geq \gamma_{threshold}$ for any successful communication. At this point, the link $l_{i}$ capacity $c_{i}$ can be given by the Shannon formula, as shown in equation (4).


$$c_{i}=B\times {log}_{\text{2}}(1+\gamma_{i})$$ (4)

In equation (4), B is the system bandwidth. The total capacity $C_{T}$ of the network during its lifetime T is defined as the sum of the amount of data successfully transmitted by all links in all time slots, and the calculation method is shown in equation (5).


$$C_{T}=\sum_{i=1}^{N}\sum_{t=1}^{T}(c_{i}^{t}\times t_{slot})$$ (5)

In this article, all “network capacity” refers to the total amount of data successfully transmitted by all links within the network life cycle T, with the unit being kilobits (kb). This value is accumulated from the link capacity $c_{i}$ of each time slot. At a 5kHz system bandwidth, a single link can achieve a transmission capacity of several kb per time slot through efficient modulation and coding strategies. Underwater nodes are powered by batteries with limited capacity. The total energy consumption $E_{i}^{t}$ of underwater sensor nodes mainly consists of four parts: transmission energy consumption $E_{\text{trans}}^{t}$, idle monitoring static power consumption $E_{\text{idle}}^{t}$, AI algorithm operation power consumption $E_{\text{AI}}^{t}$, and sensing task energy consumption $E_{\text{sense}}^{t}$.


$$\left\{ \begin{array}{c} @l@E_{i}^{t}=E_{\text{trans}}^{t}+E_{\text{idle}}^{t}+E_{\text{AI}}^{t}+E_{\text{sense}}^{t}\mathrm{\backslash vspace}1mm\\ E_{\text{trans}}^{t}=p_{i}^{t}\times t_{\text{trans}}\mathrm{\backslash vspace}1mm\\ E_{\text{idle}}^{t}=V\times I_{\text{idle}}\times (t_{\text{slot}}-t_{\text{trans}}-t_{\text{scnse}})\mathrm{\backslash vspace}1mm\\ E_{\text{AI}}^{t}=E_{\text{AI},\text{single}}\times N_{\text{infer}} \end{array}\right.$$ (6)

In equation (6), $t_{\text{trans}}$ represents the actual emission time within the current time slot. V and $I_{\text{idle}}$ represent the hardware voltage and current of the sensor node, respectively. $t_{\text{scnse}}$ represents the time consumption of the sensing task within the current time slot. $E_{\text{AI},\text{single}}$ represents the energy consumption for a single inference. $N_{\text{infer}}$ represents the number of inferences within the current time slot. The cumulative energy consumption of the node should satisfy equation (7).


$$E_{i}\left(T\right)=\sum_{t=1}^{T}e_{i}^{t}\leq E$$ (7)

In equation (7), the network lifetime T satisfies $T=\underset{i\in 𝒩}{min}T_{i}$. Underwater applications usually have a minimum requirement for network lifetime $T_{threshold}$, so it must met $T\geq T_{threshold}$. Therefore, the performance optimization problem of UWSNs can be formalized as a Dec-POMDP process. Each sending node $n_{i}$ in the network is regarded as an agent. The global state $s_{t}$ includes the position of all nodes, the remaining energy, the current channel conditions, and the actions taken by all agents. Due to the long latency of the underwater acoustic channel and the limited perception range of the nodes, the state is partially observable for a single agent [22,23].

Each agent selects a power level $a_{i}^{t}$ from a discrete set ${𝒜}_{i}=\{P_{\text{1}},P_{\text{2}},...,P_{max}\}$ of transmit powers for transmission. Selecting zero power means no transmission in that time slot. All agents share a common team reward $r^{t}$. The optimization goal is to achieve fair reuse, that is, to jointly optimize network capacity and communication fairness while meeting the survival time requirement. To comprehensively evaluate the network’s throughput efficiency and resource allocation fairness, this study defines a composite Index - Fair Reuse Index (FRI) [24]. This metric is the product of the average network reuse time (measuring the concurrent transmission capacity of the network) and the Jain fairness index (measuring the fairness between nodes), calculated in equation (8).


$$FRI=\overset{―}{u}\times F^{T}$$ (8)

In equation (8), $\overset{―}{u}$ represents the average number of successful concurrent communications per time slot during the lifetime of the network, and its value can be greater than 1, reflecting the network’s spatial multiplexing capability. $F^{T}$ is the Jain fairness index within the network lifetime, which is used to measure resource allocation fairness. $F^{T}$ ranges from 0 to 1, with values closer to 1 indicating greater fairness and closer to 0 indicating less fairness, as shown in equation (9).


$$F^{t}=\left\{ \begin{array}{c} @l@\frac{{\left(\sum_{i=1}^{N}u_{i}\right)}^{\text{2}}}{N\text{\textbackslash{}hspace\{0.33em}}\sum_{i=1}^{N}u_{i}^{\text{2}}\},\text{\textbackslash{}hspace\{0.33em}\;\;\;\;\}\sum_{i=1}^{N}u_{i}\geq 0\\ 0,\text{\textbackslash{}hspace\{0.33em}\;\;\;\}\sum_{i=1}^{N}u_{i}=0 \end{array}\right.$$ (9)

In equation (9), $u_{i}$ represents the actual amount of resources obtained by the i th underwater node. In summary, the performance optimization problem of UWSNs can be formulated as: Under the premise of satisfying energy and communication quality constraints, find a distributed strategy π, so that each agent selects the transmission power based on its local observation history $\tau_{i}^{t}$ to maximize the long-term expected cumulative reward $𝔼\left[\sum_{t=1}^{T}\gamma^{t-1}r^{t}\right]$. γ is the discount factor. The complexity of this problem lies in the fact that the agents need to achieve global collaboration through distributed decision-making in a partially observable, highly dynamic, and resource-constrained environment.

Considering the long propagation latency (typically on the order of 10^−1^ to 10^1^ s) and dynamic topological characteristics of underwater acoustic channels, perfect time synchronization cannot be achieved in engineering. Therefore, this study supplements the distributed time synchronization mechanism and models the impact of synchronization errors on time slot scheduling, making the system model more in line with the actual deployment scenarios. The distributed time synchronization scheme adopts a bidirectional synchronization protocol for underwater acoustic signals. The core logic is as follows: Each node periodically (every 5 time slots) sends synchronous beacon signals to neighboring nodes, and the signals contain the local timestamp of the sending node (accurate to 1ms). The receiving node records the signal arrival timestamp. Combined with the known estimated value of link propagation latency (obtained from previous sequence CSI and distance calculations), the time deviation between the local time and the sending node is calculated. Nodes weight and average the deviation values of multiple adjacent nodes (the weight is the link signal-to-noise ratio, the higher the signal-to-noise ratio, the greater the weight), and dynamically correct the local clock to achieve distributed time synchronization.

The synchronization error ε is defined as the time difference between the local time slot of the node and the global time slot of the network, which follows a normal distribution $\varepsilon \sim \;N\left(0,\sigma^{\text{2}}\right)$, where σ is the standard deviation of the error, with a value range of 0.1 to 0.5 seconds. If ε ≤ 2 seconds (protection interval), data transmission is normal. If ε > 2s, it is determined as a transmission failure, included in the packet loss rate, and the node is triggered to resynchronize simultaneously. Due to synchronization errors, the actual sending/receiving time of nodes may deviate from the global time slot window, which may cause signal collisions or reception failures. Therefore, in this study, the core transmission window and protection interval are designed for the time slot window. The core transmission window is 8s (accounting for 80% of the time slot length), used for data transmission. A protection interval of 2s (accounting for 20% of the time slot length) is used to counteract the time offset caused by synchronization errors and prevent signal overlap in adjacent time slots.

### 3.2. MARL-based optimization framework

**Fig 3:**
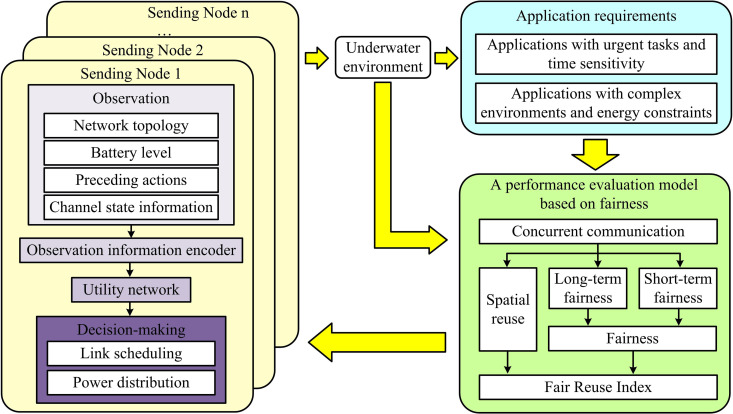
Workflow diagram of MARL.

To solve the formalized Dec-POMDP problem, this study proposes a distributed network performance optimization algorithm framework based on MARL. The core idea of this framework is to treat each underwater transmitting node as an intelligent agent. Through trial and error in a dynamic environment, it learns how to adjust transmit power based on local observations, ultimately collaboratively maximizing fair reuse in the network. The workflow of the proposed Deep Multi-agent Power Management (DMPM) framework is shown in Fig 3.

In Fig 3, the performance evaluator is responsible for selecting appropriate fairness evaluation models (short-term fairness or long-term fairness) based on application requirements such as task urgency or energy constraints. The FRI of the current network state is calculated and fed back to the system as a team reward signal. The resource manager is the core of intelligent decision-making. It receives local observation information from each node and the rewards calculated by the evaluator, and then generates power allocation decisions for each node. All agents learn collaboratively through shared reward signals during the centralized training phase, aiming to maximize the common long-term team reward, thereby demonstrating efficient collaboration during the distributed execution phase.

In the MARL framework, the action space is discrete and finite. Each action $a_{i}^{t}$ of the agent $n_{i}$ is to select a power value from a discrete set $𝒫=\{P_{\text{1}},P_{\text{2}},...,P_{\left|𝒫\right|}\}$ of transmit power levels. This set includes a zero-power option, allowing the node to voluntarily abstain from transmission in a given time slot. This decision can be seen as providing a “soft scheduling” suggestion to the underlying Medium Access Control (MAC) protocol, reducing channel competition pressure and minimizing potential conflicts by actively avoiding sending when channel conditions are poor or energy is insufficient, thus forming a synergy with the MAC layer [25,26]. However, this framework does not replace the conflict resolution mechanisms based on competition or scheduling in traditional MAC layer protocols (such as RTS/CTS or TDMA), but rather provides forward-looking assistance through intelligent power decision-making. Specifically, the framework, through MARL learning, enables nodes to proactively adjust the transmission power or choose silence before potential conflicts occur, thereby reducing the probability of conflicts in concurrent transmissions. For instance, when a node senses strong channel interference or insufficient energy of its own, its “zero-power” decision actually avoids a potentially failed transmission attempt, reducing the burden of subsequent MAC layer conflict detection and retransmission. This predictive avoidance complements the real-time conflict resolution at the MAC layer: the former reduces the frequency of conflicts from the source by learning to optimize the transmission timing and power; The latter, on the other hand, recovers in accordance with the established agreement when a conflict occurs. The synergy of the two enables the network to not only take advantage of the global benefits of intelligent optimization in shared channels but also rely on mature mechanisms to ensure transmission reliability.

The study models the power allocation problem of UWSN as a partially observable distributed Markov decision process, which can be defined as tuple $\langle 𝒩,𝒮,\left\{{𝒜}^{i}\right\},\left\{{𝒪}^{i}\right\},P,R,\gamma \rangle$. 𝒩={1,…,N} represents the set of agents (sending nodes). The state space 𝒮 is the global state $s_{t}=\left({𝐩}_{t},{𝐞}_{t},{𝐆}_{t},{𝐚}_{t-1}\right)$, which includes all node positions ${𝐩}_{t}=(p_{t}^{\text{1}},\ldots ,p_{t}^{N+M})$, residual energy ${𝐞}_{t}=(e_{t}^{\text{1}},\ldots ,e_{t}^{N})$, the current channel gain matrix ${𝐆}_{t}$, and the joint action ${𝐚}_{t-1}$ of all agents in the previous time slot. The action space ${𝒜}^{i}$ represents the action of each agent i. $a_{i}^{t}$ represents a power level from its discrete transmission power set 𝒫={0,2,4,8,16,32,64}. Selecting 0 indicates remaining silent in this time slot. The observation space ${𝒪}^{i}$ represents the local observation $o_{t}^{i}=\left(\begin{array}{c} @c@{𝒩}_{\text{local}}^{i}\left(t\right),e_{t}^{i},{\hat{g}}_{i,r}\left(t\right),(a_{t-K}^{i},\ldots ,a_{t-1}^{i}nonumber \end{array}\right)$ obtained by each agent i in time slot t. It includes: local topological information ${𝒩}_{\text{local}}^{i}\left(t\right)$ related to its own communication; Self-residual energy $e_{t}^{i}$; Local channel state information (CSI), including the estimated channel gain ${\hat{g}}_{i,r}\left(t\right)$ to its receiving end. The history of one’s previous K -step movements is $\left(a_{t-K}^{i},\ldots ,a_{t-1}^{i}\right)$. P is the state transition function, R is the team reward function, and γ is the discount factor.

The reward function is key to guiding the agent to learn the desired behavior. Therefore, this study designs a comprehensive team reward function $r^{t}$, which consists of two parts: fair reuse reward $r_{FRI}^{t}$ and survival time penalty $r_{lifetime}^{t}$, as shown in equation (10).


$$r^{t}=r_{FRI}^{t}+r_{lifetime}^{t}$$ (10)

$r_{FRI}^{t}$ is directly related to the core optimization goal of this study, as shown in equation (11).


$$r_{FRI}^{t}=l^{t}\times F^{T}$$ (11)

In equation (11), $l^{t}$ is the number of successful concurrent communications in the slot t network. This design encourages agents to increase the number of concurrent communications while also ensuring fairness in communication opportunities for all nodes. As a hard constraint, $r_{lifetime}^{t}$ ensures that the network meets the survival time requirement. Its design is shown in equation (12).


$$r_{lifetime}^{t}=\left\{ \begin{array}{c} @l@-100,\text{\textbackslash{}hspace\{0.33em}\;\;\;\}if\text{\textbackslash{}hspace\{0.33em}\}t<T_{threshold}\text{\textbackslash{}hspace\{0.33em}\}and\text{\textbackslash{}hspace\{0.33em}\}\exists i\in 𝒩,E_{i}^{remain}<E^{th}\\ 0,\text{\textbackslash{}hspace\{0.33em}\;\;\}otherwise \end{array}\right.$$ (12)

In equation (12), when the remaining energy $E_{i}^{remain}$ of any node falls below the safety threshold $E^{th}$ before the network’s required survival time $T_{threshold}$ expires. All agents receive a significant negative reward (penalty) and terminate the current training round early. This forces the agents to learn energy-saving strategies to avoid premature death of any node.

**Fig 4:**
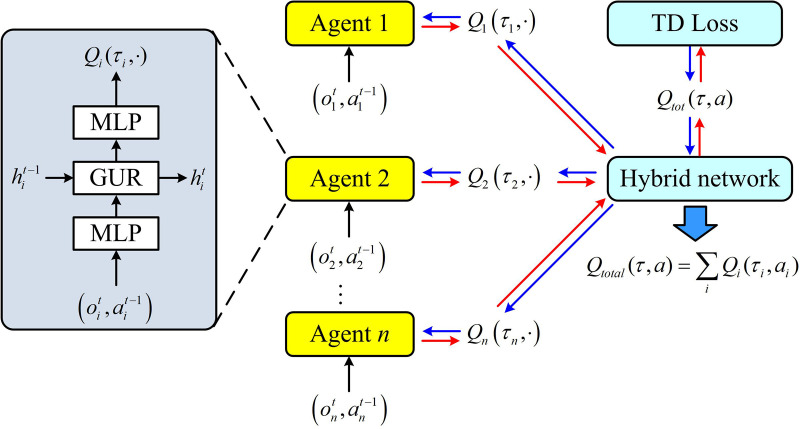
Training and distributed execution architecture.

To handle the partial observability and achieve effective collaborative learning, this study adopts a centralized training and distributed execution approach. The architecture of this mechanism is shown in Fig 4. The core idea is to use global information to learn a better strategy during training. Each agent relies only on local information to make independent decisions during execution.

In Fig 4, during the training phase, a virtual central critic or hybrid network is introduced. This network is able to obtain the local observation history $\tau =(\tau_{\text{1}},...,\tau_{N})$ and joint action a of all agents and estimate a global state-action value function $Q_{tot}\left(\tau ,a\right)$. This study adopts a linear additive method similar to VDN for value decomposition. A linear structure is selected based on two considerations: Firstly, the computational and communication overheads of linear decomposition are significantly lower than those of nonlinear hybrid networks (such as QMIX), making it more suitable for underwater nodes where computing and energy resources are strictly limited. Secondly, the linear decomposition satisfies the sufficient but not necessary conditions of the individual global maximum (IGM) principle, theoretically ensuring that distributed strategies can effectively approach centralized optimal solutions in partially observable and medium-sized agent scenarios. The global Q-value is the linear sum of the Q-value of individual agents, as shown in equation (13).


$$Q_{tot}\left(\tau ,a\right)=\sum_{i=1}^{N}Q_{i}(\tau_{i},a_{i};\theta_{i})$$ (13)

In equation (13), $Q_{i}$ is the individual Q-network of the agent i, and $\theta_{i}$ is its network parameter. By minimizing the global temporal difference error through backpropagation, the parameters of all individual Q-networks can be updated simultaneously. The experience replay mechanism and the target network are used to stabilize the training process [27]. After the training is completed, the central critic and the hybrid network are removed. Each agent only retains its trained individual Q-network $Q_{i}\left(\tau_{i},a_{i}\right)$. In actual deployment, each node selects the action (transmission power) that maximizes the $Q_{i}$ value according to its current local observation history $\tau_{i}^{t}$ through a greedy strategy, as shown in equation (14).


$$a_{i}^{t}=arg\underset{a\in {𝒜}_{i}}{max}Q_{i}\left(\tau_{i}^{t},a\right)$$ (14)

**Fig 5:**
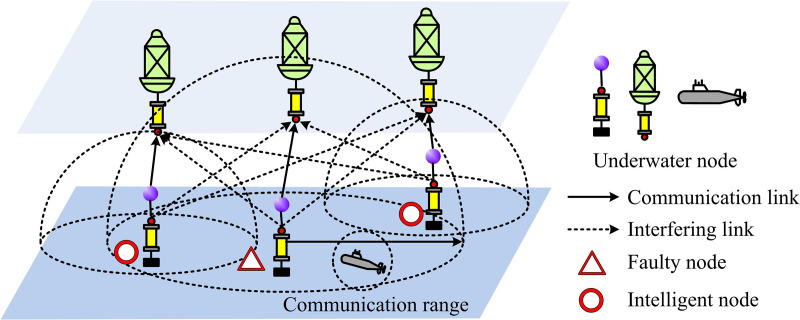
UWSN communication scenarios including node failures.

This mechanism perfectly matches the distributed nature of UWSNs. The decision-making process does not require real-time communication between nodes, avoiding the effects of additional channel overhead and long latency. The study also considers an imperfect UWSN, as shown in Fig 5.

**Fig 6:**
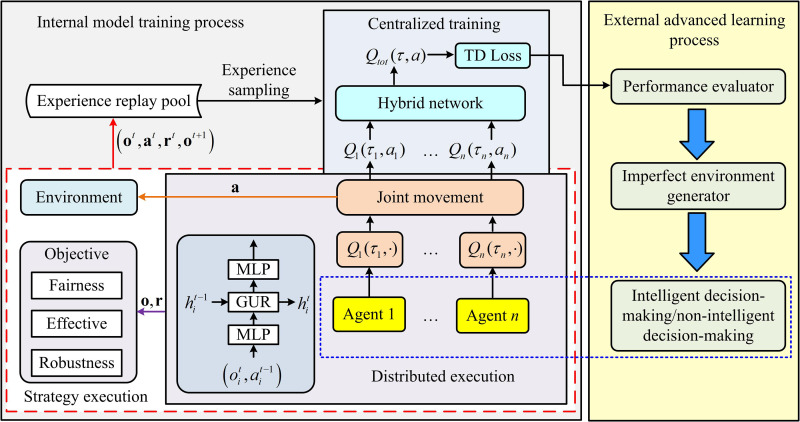
Process of semi-cooperative power allocation mechanism.

In real-world UWSN deployments, nodes often experience unexpected failures due to hardware aging, software anomalies, or environmental degradation, resulting in a non-ideal network environment where rational and faulty nodes coexist. To improve the network’s adaptability under non-ideal conditions, this study introduces a semi-cooperative power allocation mechanism into the existing MARL framework. The core idea of this mechanism is shown in Fig 6.

In Fig 6, by simulating node failure behavior, the agent gains the ability to identify and avoid irrational interference. Each time slot node i enter the failure mode with a probability $p_{f}$. Once a fault occurs, its action $a_{i}^{t}$ is no longer generated by its strategy $\pi^{i}$, but follows a preset fault behavior distribution $\mu_{f}$. During training, the agent’s observation $o_{t}^{i}$ additionally includes a binary fault identifier $f_{t}^{i}$ and an estimated abnormal interference from the local CSI, which is used to learn to identify interference caused by potential faulty nodes. Based on the team reward $r^{t}$, to encourage the agent to actively avoid the interference caused by faulty nodes, a local avoidance reward item $r_{t}^{avoid,i}$ is added. This reward is only used during the training phase to prompt the agent to reduce the transmission power or adjust the timing when it senses abnormally high interference. After the training is completed, the fault behavior model $\mu_{f}$ and the additional reward items are removed. Each deployed node only uses its learned strategy $\pi^{i}$ to make power decisions based on local observations, without the need to identify or communicate the status of other nodes online, thus achieving fully distributed and robust operations.

The MARL power management framework proposed in this paper operates at a higher level of the network protocol stack. Its decision (power level/transmission silence) directly affects the SINR at the physical layer and the effective data transmission at the link layer. A higher SINR means an increase in the success rate of data frame transmission and a reduction in re-transmission for the underlying MAC protocols (such as TDMA slots or competitive protocols), which is reflected in an increase in end-to-end throughput, a reduction in latency, and an improvement in energy efficiency at the network layer. The training process is shown in Algorithm 1.

## Algorithm 1

Input: Environment ε, number of agents N, total training episodes E, experience replay buffer capacity D, batch size B, discount factor γ, exploration rate decay schedule $\epsilon_{\text{decay}}$, target network update frequency C.

Output: Trained individual Q-network parameters $\{\theta_{i}{\}}_{i=1}^{N}$.

1: Initialize individual Q-network parameters $\theta_{i}$ and target network parameters $\theta_{i}^{-}\leftarrow \theta_{i}$ for all agents.

2: Initialize experience replay buffer𝒟←∅, global training step counter $t_{\text{global}}\leftarrow 0$.

3: for episodee=1 to E do

4:   Reset environment, obtain initial observations $o_{i}\leftarrow o_{\text{0}}^{i},\forall i\in \{1,\ldots ,N\}$.

5:   Initialize GRU hidden states $h_{i}\leftarrow 0$ for each agent.

6:   for time slot t=1 to T do

7:     for each agent i=1 to N do

8:       With probability ϵ select a random action $a_{i}^{t}$, otherwise select $a_{i}^{t}=arg\underset{a^{\prime }}{max}Q_{i}(\tau_{t}^{i},a^{\prime };\theta_{i})$.

9:     end for

10:    Execute joint action ${𝐚}_{t}=(a_{t}^{\text{1}},\ldots ,a_{t}^{N})$. Environment transitions to new state $s_{t+1}$, yields team reward $r_{t}$, and each agent receives new observation $o_{t+1}^{i}$.

11:    Store experience tuple $\left(s_{t},{𝐚}_{t},r_{t},s_{t+1}\right)$ into 𝒟.

12:    if |𝒟|≥B then

13:      Randomly sample a batch of B experiences from 𝒟.

14:      for each sampled experience do

15:        Compute target global Q-value: $y=r+\gamma \cdot Q_{\text{tot}}\left(s^{\prime },{𝐚}^{\prime \text{argmax}};\left\{\theta_{i}^{-}\right\}\right)$ where $\begin{array}{c} {{𝐚}^{\prime }}^{\text{argmax}}=\left(arg\underset{a^{\prime }}{max}Q_{\text{1}}(\tau^{{\text{1}}^{\prime }},a^{\prime };\theta_{\text{1}}),\ldots ,arg\underset{a^{\prime }}{max}Q_{N}(\tau^{N^{\prime }},a^{\prime };\theta_{N})\right) \end{array}$.

16:      end for

17:      Compute loss: $\begin{array}{c} ℒ\left(\theta \right)=\frac{\text{1}}{B}\sum_{j=1}^{B}{\left(y_{j}-Q_{\text{tot}}\left(s_{j},{𝐚}_{j};\left\{\theta_{i}\right\}\right)\right)}^{\text{2}} \end{array}$

18:      Update all individual Q-network parameters $\theta_{i}$ using the Adam optimizer.

19:      if $t_{\text{global}}$ mod C=0 then

20:        Update target network parameters: $\theta_{i}^{-}\leftarrow \theta_{i}$.

21:      end if

22:       $t_{\text{global}}\leftarrow t_{\text{global}}+1$

23:    end if

24:    Update each agent’s observation-action history: $\tau_{t+1}^{i}\leftarrow \left(\tau_{t}^{i},a_{t}^{i},o_{t+1}^{i}\right)$.

25:  end for

26:  Update exploration rate: $\epsilon \leftarrow \epsilon \cdot \epsilon_{\text{decay}}$

27: end for

### 3.3. Implementation in Aqua-sim platform

To achieve end-to-end multi-hop communication, this study designs a MARL-driven distributed multi-hop routing mechanism that deeply collaborates with power control and conflict avoidance mechanisms. The core includes three key modules: path discovery, next-hop selection, and data relay. The distributed path discovery mechanism does not require global topological information and dynamically constructs paths based on local observations: After a node starts, it periodically broadcasts the “Path Probe Packet” (PPP), which contains its own ID, water depth level, remaining energy and the list of discovered upper-level nodes. After receiving the PPP from the neighboring nodes, the node screens the candidate next-hop set that meets the conditions and stores it in the local “routing table”. If no candidate next hop is found, the node will increase the transmission power to expand the detection range. Meanwhile, a “routing request” is sent to the nodes at the same level. The upper-level nodes will be detected through the relay of the nodes at the same level to avoid path breakage.

In the MarL-driven next-hop selection strategy, each node acts as an Agent and incorporates “next-hop selection” into the action space, forming a collaborative optimization with the transmission power decision: The action $a_{i}^{t}$ of the Agent consists of two parts, one is transmission power selection, and the other is next-hop selection. Routing decisions are based on the routing-related dimensions observed locally, including the remaining energy of the candidate next hop, link quality, historical forwarding success rate, and the distance from the hop count to the gateway. The optimal next hop is output through the Q network. If the selected next-hop node fails (does not respond to RTS/CTS), the Agent immediately selects the suboptimal next-hop from the routing table and simultaneously triggers PPP re-transmission to update the routing table, achieving path fault tolerance.

Data relay and energy balance design are mainly to prevent relay nodes from excessive energy consumption. It achieves synergy through routing reward design and energy state adaptation: When an agent forwards data from lower-level nodes and successfully passes the data to the next hop, it can receive an additional reward, which is linked to the fair reuse index to encourage efficient relay behavior. If the remaining energy of the relay node is less than 5,000J, its routing priority will automatically decrease. When neighboring nodes choose the next hop, they will actively avoid this node. Meanwhile, this node will send an “energy alert” to nodes at the same level to guide data diversion. The data packet carries a “hop count counter” and a “check code”. Before forwarding, the relay node will verify the data integrity. If the data is lost, it will trigger re-transmission to ensure the reliability of end-to-end communication.

To cope with the dynamics and engineering constraints of real underwater environments, this study implements the proposed system model and mobility modeling method in the commonly used underwater sensor network simulation platform Aqua Sim. Aqua Sim native only supports random walk movement models and cannot accurately simulate the controlled drift behavior of anchor nodes. Therefore, this study extends its mobile model library and implements a three-dimensional mobile model that conforms to the motion characteristics of real underwater anchor nodes. The specific steps are as follows:

(1) Node class extension: Add variables locX, locY, and locZ in undersea sensor node. h to record the initial static position of the node, and add the version variable to mark the position update status.
(2) Topology enhancement: Add a variable in ns_2.3/common/topology. alpha represents the maximum offset angle of the anchor node (angle with the vertical direction). Theta represents the rotation angle between the projection of the anchor line on the horizontal plane and the true north direction. Version is the global version number used to synchronize the movement status updates of all nodes. Time_ handler represents a timer that controls the update cycle of the movement state. After deployment, the timer cycle is triggered to randomly generate new theta angles, increment the global version, and reset the timer.
(3) Implement anchor node mobility model: Create a new archor_madel.cc under ns_2.3/underwater sensor/uw-mobilility_model/and implement the update_position () function. This function reads theta, alpha, and the current version number from the topology, and combines them with the randomly generated offset angle to calculate the new position of the node according to equation (3), simulating the restricted motion under the action of water flow.
(4) Distributed node location synchronization mechanism: All nodes share the same topology object and are bound to an archor_madel. The node periodically checks the global version locally. If a version update is found, it calls its own update_position () to update the position, ensuring that the movement of nodes within the topology has spatial correlation and conforms to the consistency and coordination of real underwater flow.

## 4. Results and analysis

The study conducted testing and evaluation through multiple sets of simulation experiments, focusing on the model’s optimization capability in distributed power management strategies and the adaptability of semi-cooperative mechanisms in networks with faulty nodes.

### 4.1. MARL model performance analysis

Experiments were conducted to construct two typical UWSN communication scenarios: a homogeneous network scenario (HomNet) and a heterogeneous network scenario (HetNet). In HomNet, all nodes are fixed sender-receiver pairs, sharing the same communication resources. The algorithm needs to learn how to efficiently compete for and cooperate among these nodes. HetNet, based on the homogeneous network, introduces one or more mobile nodes. These mobile nodes traverse the network area via random paths. When they approach a fixed receiver node, they cause significant, time-varying interference to its communication link.

To construct a time-varying underwater acoustic channel that conforms to the physical characteristics of the real ocean, this study utilizes the monthly average temperature and salinity profile data from the Argo Real-time Data Center in China (starting from January 2004, covering 180°W to 180°E, 80°S to 80°N, with a horizontal resolution of 1° × 1°, 58 standard vertical layers, and a depth of 0–1,975m). The experimental area is selected near the equator of the Pacific Ocean (165.5°-179.5°E, 0.5°N-9.5°N), and it is converted into time-varying channel gain through the systematic acoustic modeling process. Firstly, the study uses the Chen-Millero empirical formula to calculate the temperature, salinity and depth information in the Argo data as the sound velocity profile. Secondly, the calculated sound velocity profile is input into the ray-acoustic model (Bellhop model) to simulate the propagation path of sound waves from the transmitting node to the receiving node. Finally, the comprehensive time-varying channel gain is obtained by integrating the propagation loss calculated by the ray model, the frequency-dependent absorption loss (calculated using the Thorp formula), and the lognormal fading simulated by small-scale random fluctuations. Model training was implemented using TensorFlow/Keras and PyTorch, using an NVIDIA GTX 1080Ti GPU and an Intel Core i7 7800X CPU. The core simulation parameters are shown in Table 1.

**Table 1:** Parameter settings.

Parameter category	Parameter	Value
Network deployment	Fix the number of sender – receiver node pairs	{2, 3, 4, 5, 6}
	Number of mobile nodes (HetNet only)	1
Energy and time	The initial battery capacity of the fixed node	20,000 J
	The minimum required network lifetime	60 hours slot
	Single time slot length	10 s
Communication parameters	The set of available transmission power for nodes	{0, 2, 4, 8, 16, 32, 64} W
	Mobile node transmission power (fixed)	16 W
	Carrier center frequency	25 kHz
	System bandwidth	5 kHz
MARL training parameters	Discount factor	0.9
	Exploration factor	It linearly decays from 1 to 0.05
	Capacity of the experience replay pool	10000
	Small batch sampling size	32
Q Network	Input layer	Input dimension: 16 dimensions; Output dimension: 16 dimensions
	GRU layer	Input dimension: 16 dimensions; Output dimension: 32 dimensions
	Fully connected layer 1	Input dimension: 32 dimensions; Output dimension: 64 dimensions
	Fully connected layer 2	Input dimension: 64 dimensions; Output dimension: 32 dimensions
	Output layer	Output dimension: 32 dimensions
	Activation function	All fully connected layers use the ReLU activation function
Network deployment	Deployment area	2 km × 2 km × 200 m (length×width×depth)
	Fixed node spacing	300-500 m (randomly and uniformly distributed)
	Mobile node path	Random waypoint model
Sound propagation model	Deterministic model	Bellhop ray tracing
	Absorption-loss model	Thorp formula
	Decline model	Lognormal fading
Noise model	Environmental noise power spectral density	50 dB re μPa/Hz
Simulation engine	Simulation time step	10 s
	Total simulation duration	1,000 time slots
	Simulation platform	Aqua-Sim

The performance indicators used for evaluation in the research are defined as follows: “Network multiplexing” refers to the average number of links successfully established for concurrent communication within each time slot in the network, with the unit being “number of links/time slot”. “Concurrency” is the ratio of the actual number of successful concurrent links to the theoretical maximum possible number of links under the current topology, which is a dimensionless proportional value (ranging from 0 to 1). As defined in equation (5) mentioned earlier, “network capacity” refers to the total amount of data successfully transmitted during the network’s lifetime, measured in kb.

**Fig 7:**
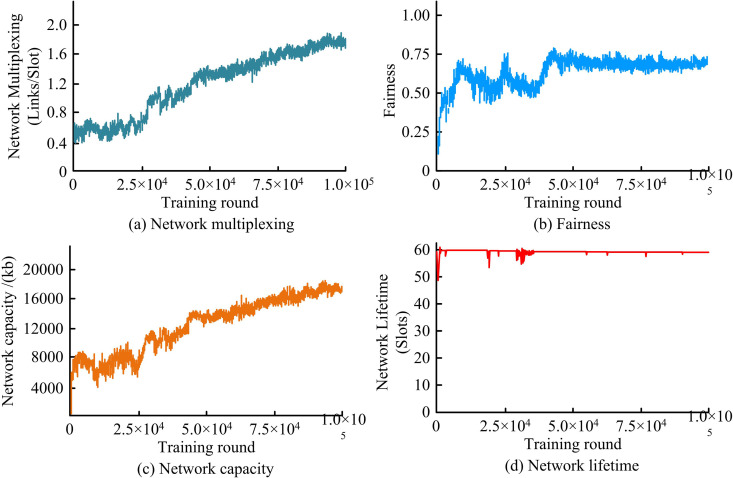
Network performance during the training phase.

Fig 7 shows how four the key metrics of network performance during the training phase change with each training round: network reuse, fairness, network capacity, and network lifetime. In Fig 7(a), the number of successful concurrent communications increased with each training round, indicating that the agent gradually learned to coordinate resources to improve reuse efficiency. In Fig 7(b), the fairness exhibited a continuous optimization trend during training, eventually reaching a stable state. Fig 7(c) showed that the network capacity increased with the number of training rounds. Fig 7(d) reflected that the agent effectively mastered the control strategy to avoid premature energy depletion of nodes through RL. Overall, all metrics showed a positive upward trend during training and reached a stable state in the later stages, demonstrating the effectiveness and convergence of the introduced MARL framework in improving overall network performance.

This study compared six power allocation methods in a HetNet scenario, with the results shown in Table 2. Fixed Power Allocation (FPA) used a continuous transmission power of 16W. Alternating Power Allocation (APA) used a time-slotted transmission power of 16W. Random Power Allocation (RPA) and Random Alternating Power Allocation (RAPA) demonstrated the performance of uncoordinated random strategies. COMA and the proposed MARL model represented learning-based adaptive strategies. As shown in Table 2, DMPM achieved both the optimal network capacity (24,568.3 kb) and FRI (1.85) using adaptive power allocation. This method achieved a 5.3% capacity improvement and a 9.5% FRI improvement over the second-best performing COMA method. While traditional methods such as APA achieved higher capacity at 16W, their FRI was only 0.58. While FPA achieved a fairness index of 0.95, it only lasted for 55 time slots due to excessive energy consumption, failing to meet the 60-time slot lifetime requirement. This result demonstrated the MARL model’s remarkable ability to collaboratively optimize network capacity, communication fairness, and lifetime in complex interference environments.

**Table 2:** Performance of the MARL model and other comparison methods.

Method	Network capacity/(kb)	FRI	Network lifetime/(time slot)	Average delivery latency/(time slot)
FPA	12580.6	0.95	45	3.8
RPA	15823.4	0.72	55	2.5
APA	18245.1	0.58	60	4.2
RAPA	16512.8	0.41	58	3.5
COMA [14]	22037.9	1.62	60	1.5
This study	24568.3	1.85	60	1.2

**Fig 8:**
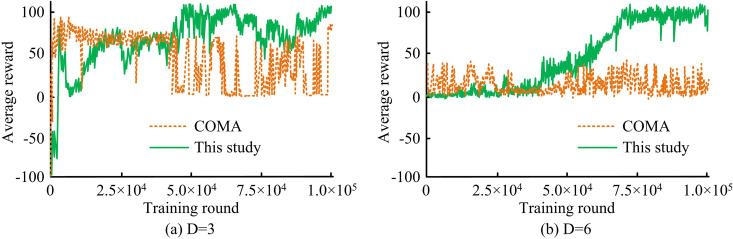
Training curves under different network densities in the HomNet scenario.

Fig 8 shows the training curves for different network densities (D = 3 and D = 6) in the HomNet scenario. In Fig 8(a), the average reward of the proposed method rapidly increased from the initial stage and eventually stabilized above 90. In Fig 8(b), the average reward of the proposed method continued to rise, eventually approaching 100. This indicated that the proposed method could effectively improve the average reward.

**Fig 9:**
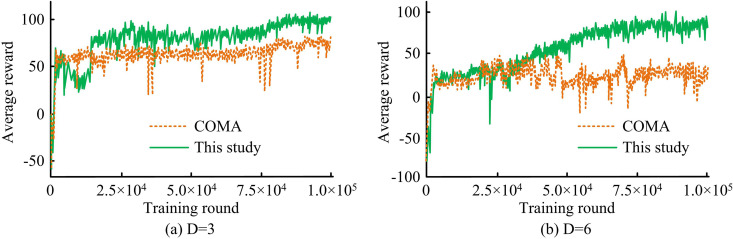
Training curves under different network densities in the HetNet scenario.

Fig 9 illustrates the training process in the HetNet scenario. In Fig 9(a), the average reward of the proposed method steadily increased from the initial stage, eventually stabilizing above 90. COMA exhibited a certain learning ability in the early stages of training, but fluctuates greatly in the later stages, and its overall performance is much lower than the proposed method. In Fig 9(b), the average reward of the proposed method maintained an upward trend, eventually approaching 100. The average reward of the COMA fluctuated around 20. These results indicated that in complex interference environments with high network density, the proposed method had better convergence characteristics and final performance than the COMA.

Table 3 shows the performance comparison results in an imperfect underwater sensor network scenario with a 5% probability of node failure. The proposed method had significant advantages in communication latency control. It maintained the lowest average latency in both low-density and high-density network configurations. In a six-node network scenario, its latency was only 6.18 time slots, while the traditional FPA method reached 60 time slots, indicating that the latter was essentially ineffective. Even the best performing benchmark algorithm COMA achieved a latency of 9.76 time slots in this case, which is nearly 0.5 times higher than the proposed method. As the network density increases from 2 to 6, the latency of the proposed method steadily increases from 1.52 time slots to 6.18 time slots. This is much smaller than the drastic fluctuation of RPA method from 4.83 time slots to 21.65 time slots. This indicates that the semi-cooperative mechanism can effectively maintain low communication latency in the dynamic environment of underwater networks by intelligently avoiding interference from faulty nodes and optimizing transmission scheduling.

**Table 3:** Average latency of MARL model and baseline methods under different network densities.

Network density	FPA	APA	RPA	COMA [14]	This study
D = 2	3.52	2.01	4.83	2.85	1.52
D = 3	12.71	3.02	7.24	4.13	2.31
D = 4	28.53	4.03	10.87	5.52	3.64
D = 5	55.14*	5.01	15.31	7.38	4.82
D = 6	60.00*	6.02	21.65	9.76	6.18

Table 4 shows the simulation results under different synchronization error levels. The low error scenario (σ = 0.1s) has a high node density (D ≥ 4), with ≥3 neighboring nodes and a synchronous beacon receiving signal-to-noise ratio of ≥ 15dB. The medium error scenario (σ = 0.3s) has a medium node density (2 ≤ D ≤ 3), with=2 neighboring nodes and a synchronous beacon receiving signal-to-noise ratio of 10 ~ 15dB. The high error scenario (σ = 0.5s) has a low node density (D = 2), with=1 neighboring node and a synchronous beacon receiving signal-to-noise ratio of<10dB. As shown in Table 4, an increase in synchronization error leads to a decrease in the performance of all algorithms, but the attenuation amplitude of the proposed algorithm is the smallest. In high error scenarios, the network capacity of the proposed algorithm only decreased by 10.5% compared to low error scenarios, while COMA decreased by 12.7% and FPA decreased by 10.4%. The packet delivery rate of the proposed algorithm only decreased by 4.5%, far lower than that of COMA (7.9%) and FPA (7.2%), indicating its stronger robustness to synchronization errors. The proposed algorithm perceives the risk of transmission failure in real time through local observation. If a large synchronization error is detected (estimated by the time deviation of the beacon signal), the transmission power will be actively reduced to reduce the signal coverage range, avoid asynchronous signal collisions with other nodes, and extend the data re-transmission interval to balance latency and reliability.

**Table 4:** Simulation results under different synchronization error levels (Shallow sea complex channel, D = 4).

Step error grade	Algorithm	Network capacity/(kb)	Average latency/(time slot)	packet delivery rate (%)	Energy efficiency/(kb/J)
Low error (σ =0.1s)	FPA	8321.5	17.8	69.1	0.22
	COMA [14]	17056.2	8.5	87.3	0.34
	This study	19428.7	5.4	92.1	0.39
Mean error (σ =0.3s)	FPA	7985.3	19.2	66.5	0.2
	COMA [14]	16024.8	9.8	83.7	0.32
	This study	18635.2	6.1	90.2	0.37
High error (σ =0.5s)	FPA	7458.6	22.3	61.9	0.18
	COMA [14]	14892.5	11.5	79.4	0.29
	This study	17382.4	7.3	87.6	0.34

### 4.2. Performance of MARL model in underwater environment

**Fig 10:**
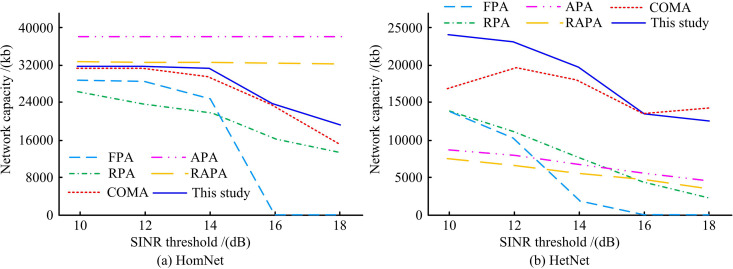
The influence of SINR thresholds on network performance in various scenarios.

Fig 10 illustrates the impact of SINR thresholds on network performance in various scenarios. In Fig 10(a), the network capacity of the proposed method remained relatively stable at around 32,045Kb. In Figure 10(b), the proposed method maintained a leading advantage in network capacity throughout. The proposed method could maintain a high network capacity in various test scenarios. In contrast, other comparative algorithms were more significantly affected by the SINR threshold, with greater performance degradation. This is because when the SINR threshold rises, the intelligent system will be more inclined to allocate higher power to the links with better channel conditions to ensure successful communication, while making the links in deep fading actively silent to avoid energy waste and interference. This intelligent power adjustment and silence mechanism based on local observation enables the network capacity to remain stable even under high SINR requirements, and the attenuation is much smaller than that of the comparison algorithm. This demonstrates the strong adaptability of the MARL strategy to maintain the overall network throughput rate under dynamic threshold constraints.

**Fig 11:**
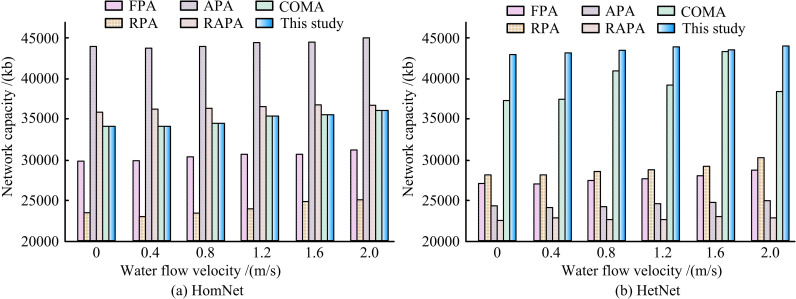
The influence of water flow velocity on network performance in different scenarios.

Fig 11 indicates the impact of water flow velocity on network performance in different scenarios. In Fig 11(a), the network capacity of the proposed method remained stable and high as water flow velocity changed. At a water flow velocity of 2.0 m/s, it still performed well. While algorithms such as APA initially had high network capacity, their stability was insufficient. In Fig 11(b), the network capacity of the proposed method was significantly higher at all water flow velocities. For example, at water flow velocities of 0 and 0.4 m/s, the network capacity exceeded 40,000 Kb, and the attenuation with increasing water flow velocity was much smaller than that of other algorithms. The network capacity such as FPA and RPA was significantly lower, and fluctuated significantly with changes in water flow velocity. The fundamental reason lies in the fact that the proposed tethered node movement model was integrated into the training environment of MARL, enabling the agent to come into contact with and adapt to the continuous and correlated topological dynamics caused by water flow during the learning stage. The strategy learned by the agent not only relies on instantaneous distances but also can infer the movement trends of nodes from the observed history, thereby making more forward-looking power decisions. In contrast, the baseline algorithm does not embed an understanding of such related movements, so its performance is more sensitive to changes in flow velocity and fluctuates sharply.

**Fig 12:**
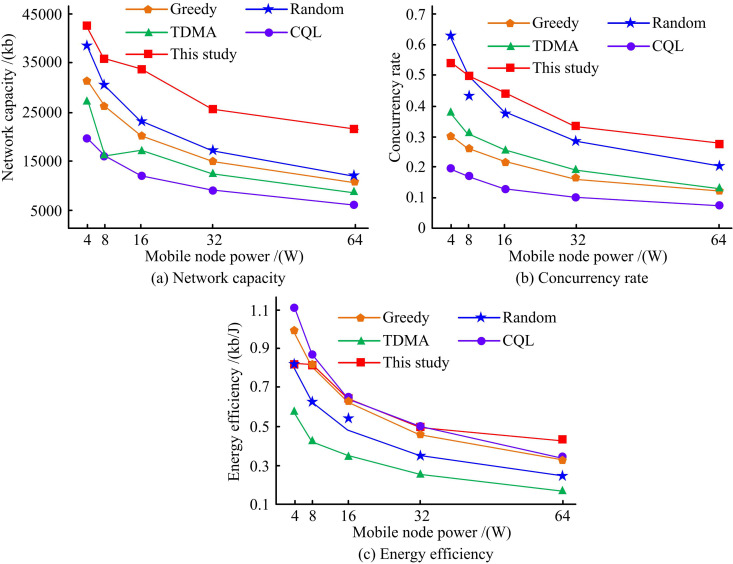
The impact of mobile node interference on network performance.

Fig 12 illustrates the impact of mobile node interference on network performance. The Greedy scheme, Random, Time Division Multiple Access (TDMA), Collaborative Q-learning (CQL)-based approach, and the proposed method across three dimensions: network capacity, concurrency, and energy efficiency are compared. In Fig 12(a), the network capacity of the proposed method gradually decreased with the increase of mobile node transmit power, but it always maintained a leading performance. At 4W power, the capacity was close to 35,000Kb. At 64W, it could still maintain a transmission capacity of over 20,000Kb, significantly better than other comparative schemes. The concurrency comparison in Fig 12(b) showed that the proposed method achieved a concurrency level of 0.5 at 4W and remained above 0.2 at 64W. The greedy algorithm, random strategy, and other comparative methods showed more significant decreases, and the CQL method exhibited the most limited performance. Looking at the energy efficiency index in Fig 12(c), the proposed method showed the smallest attenuation, reaching 0.8kb/J at 4W and maintaining an energy efficiency of 0.4kb/J at 64W. In contrast, the TDMA method dropped below 0.1kb/J at 64W.

The high-consumption training phase is carried out on offline servers or surface base stations with powerful computing capabilities, as underwater nodes cannot handle such continuous high-intensity operations. When each underwater node is deployed, only the parameters of the trained individual Q-network need to be loaded. During execution, the MCU of the node only needs to complete one forward propagation (inference). That is, based on the current local observation $\tau_{i}$, the Q-value of each action is calculated through the network and the power level corresponding to the maximum value is selected. To evaluate the feasibility of performing inference on a typical underwater sensor node MCU, the individual Q-network is intentionally designed as a simple-structured MLP, with the input layer being the local observation dimension and the output layer being the number of optional power levels. The number of parameters is reduced by limiting the number of network layers and neurons. To verify the hardware feasibility, the STM32L476 and Arduino Uno platforms are selected to test the lightweight model. The results are shown in Table 5. Both hardware devices meet the constraints. The inference time, memory, and energy consumption have not exceeded the upper limit, and the core performance has only slightly declined. They are fully compatible with the actual deployment of sensor nodes.

**Table 5:** Comparison of inference overhead among different micro-controller platforms.

MCU	Inference time (ms)	Memory usage (KB)	Single energy consumption (mJ)
STM32L476RG	38.2	18.5	1.9
Arduino Uno	102.7	19.3	5.1

Table 6 compares the proposed method with four modern MARL baselines in the HetNet scenario. All algorithms are based on the same environmental interface and training steps. Each algorithm runs independently under 10 different random seeds, and the results are presented as mean±standard deviation. The network capacity of the proposed method is significantly better than that of MADDPG, VDN and COMA (*p* < 0.01), and there is no significant difference compared with QMIX (*p* = 0.052). The FRI of the proposed method was significantly better than that of all baselines except QMIX (*p* < 0.05), and the marginal difference from QMIX was significant (*p* = 0.048). The average latency of the proposed method was significantly better than that of all baselines (*p* < 0.05). Specifically, compared with VDN, which also adopts linear value decomposition, the significant advantages of DMPM in capacity and fairness verify the effectiveness of the customized composite reward function. It can better guide the agent to make decisions conducive to long-term multi-objective optimization in partially observable environments than the single global reward of VDN.

**Table 6:** Performance comparison statistics results with modern MARL baselines (HetNet scenario, n = 10).

Method	Network capacity (KB)	FRI	Average delivery latency (time slot)	References
DMPM	25432.7 ± 415.3	1.91 ± 0.07	1.15 ± 0.18	This study
VDN	21023.6 ± 587.1 **	1.42 ± 0.11 **	1.88 ± 0.28 **	S. Cao et al. [11]
QMIX	24105.9 ± 498.6	1.80 ± 0.09	1.38 ± 0.21 *	R. Fan et al. [12]
MADDPG	19875.4 ± 632.8 **	1.35 ± 0.13 **	2.05 ± 0.31 **	H. Zhang et al. [13]
COMA	22547.3 ± 560.2 **	1.68 ± 0.12 **	1.52 ± 0.24 **	T. Zhang et al. [14]

Note: * indicates *p* < 0.05, and ** indicates *p* < 0.01.

Under the same Settings of the HetNet scenario and a 5% node failure rate, the DMPM ablation experiment was designed and executed. The results are shown in Table 7. Among them, DMPM-Full is the complete model proposed in this paper. DMPM-NoMove removes the passive motion model of nodes. DMPM-NoPenalty removes the lifetime hard constraint penalty term in the reward function. DMPM-NoGuard has removed the semi-cooperative mechanism. Table 7 shows that the complete model DMPM-Full significantly outperforms all ablation variants in all indicators. It has the highest network capacity, reaching 25,432.7 kilobytes, the lowest average delay, which is only 1.15 time slots, and fully meets the network lifetime requirement of 60 time slots. The average delay of DMPM-NoMove rose to 2.85 time slots, and the network capacity decreased significantly (*p* < 0.01). The average lifetime of the DMPM-NoPenalty network has dropped to 43.2 time slots, which proves the decisive role of this module in ensuring long-term operation. DMPM-NoGuard performed the worst, with an average delay as high as 4.18 time slots, and fairness was significantly reduced (*p* < 0.01), highlighting the key value of this mechanism in maintaining network robustness. Ultimately, the complete model demonstrated comprehensive and significant performance advantages over all ablation variants, verifying the effectiveness of the collaborative design of each innovative module.

**Table 7:** Results of ablation experiment.

Method	Network capacity (kb)	FRI	Average delay (time slot)	Network lifetime (time slot)	Energy efficiency (kb/J)
DMPM-Full	25432.7 ± 415.3	1.91 ± 0.07	1.15 ± 0.18	60.0 ± 0.0	0.41 ± 0.02
DMPM-NoMove	21685.4 ± 587.1 **	1.62 ± 0.11 **	2.85 ± 0.31 **	60.0 ± 0.0	0.35 ± 0.03 **
DMPM-NoPenalty	24105.8 ± 502.6 *	1.82 ± 0.09	1.42 ± 0.23 *	43.2 ± 3.5 **	0.39 ± 0.02
DMPM-NoGuard	22974.6 ± 543.2 **	1.70 ± 0.10 **	4.18 ± 0.42 **	60.0 ± 0.0	0.37 ± 0.03 *

Note: * indicates *p* < 0.05, and ** indicates *p* < 0.01.

## 5. Discussion

### 5.1. Inherent challenges and adaptive trade-offs of the MARL framework

Although the CTDE framework adopted in this study theoretically supports the synergy of global optimization and distributed decision-making, it still faces inherent challenges such as partial observability and credit assignment in actual deployment. Although the study reduced the computational and communication overhead through linear value decomposition to make it more suitable for resource-constrained underwater nodes, the monotonicity assumption of linear decomposition may limit the expressive ability of the strategy in complex interference scenarios. Compared with methods that are completely based on monotonic hybrid networks (such as QMIX), this method has some relaxation in theoretical guarantee. However, by introducing a composite reward structure of fair reuse rewards and lifetime penalties, it partially compensates for the performance loss that may be caused by insufficient capture of nonlinear relationships. It is worth noting that in scenarios where dynamic topologies coexist with node failures, agents need to rely solely on local historical observations to make decisions, which is essentially a non-stationary learning environment. The semi-cooperative mechanism models the node failure behavior as part of the environmental dynamics and introduces local avoidance rewards during the training phase, enabling the agent to learn to identify and avoid abnormal interference rather than relying on online state communication.

### 5.2. Correlation analysis between system model and robustness

The robustness claims of this study are mainly based on explicit modeling and analysis of multiple uncertainties such as node failures, synchronization errors, and hydrological dynamics. In terms of node failures, the study simulated irrational node behaviors through random fault injection (with a 5% probability) and verified the effectiveness of the semi-cooperative mechanism in maintaining low communication latency. However, the diversity of failure modes (such as permanent hardware failure, intermittent software errors or malicious attacks) may exceed the coverage of the current random failure model. For instance, the GCN-LG trust model proposed by B. Jiang et al. [28] explicitly detects and isolates malicious nodes through graph convolutional networks and trust mechanisms, while this study adopts adaptive power adjustment and avoidance strategies. The fundamental ideas of the two in dealing with node anomalies are different: The former focuses on security and attack identification, while the latter emphasizes maintaining sustained performance under interference. In terms of synchronization error, the research mitigated the impact of time slot offset by designing protection intervals and distributed synchronization mechanisms. However, the coupling relationship between error levels and network density as well as channel quality may cause the synchronization performance to deteriorate under extreme conditions.

### 5.3. Fundamental differences in problem formalization from related studies

Compared with existing studies, the core difference of this work in problem formalization lies in incorporating power allocation, fairness, lifetime and irrational interference into a unified distributed partially observable Markov decision process framework, and achieving multi-objective collaborative optimization through a composite reward function. For instance, Z. Liu et al. [29] employed deep RL for multi-UAV task allocation, focusing on task completion rate and path planning, without explicitly considering communication interference between nodes and long-term energy equity issues. The essence of the research problem by S. Kumari et al. [30] is static resource allocation rather than real-time decision-making at the dynamic communication level. In contrast, this study directly couples power control with topological dynamics (such as node drift) and captures the sustained impact of topological changes caused by water flow on channel gain through a passive motion model of anchor nodes, thereby enabling the learning strategy to adapt to hydrological dynamics.

### 5.4. Research limitations and technical sensitivity

The MARL-driven power management framework provides a feasible approach for the autonomous optimization of UWSN in dynamic and unreliable environments. Its technical path demonstrates the potential of integrating the physical layer channel model, node mobility and network layer constraints into the learning framework, which is conducive to promoting the application of cross-layer intelligent optimization in constrained networks. However, this method relies on global information for centralized training during the training stage. Although it does not affect the distributed characteristics of the execution stage, the training process needs to be completed on offline servers or surface base stations, and an accurate environmental model is required to generate training data. Secondly, this framework assumes that all nodes have isomorphic decision-making capabilities and observation dimensions. However, in actual heterogeneous networks, policy transfer and generalization may further consider the heterogeneity between the observation space and the action space. Future work can be deepened in the following aspects: Firstly, real water experiments can be conducted on actual sound channels and node hardware platforms to verify the effectiveness of simulation conclusions; Secondly, transfer learning and meta learning techniques can be explored to enable training strategies to quickly adapt to new environments or types of interference.Thirdly, it is necessary to consider the deep integration with security mechanisms, such as combining trust models or anomaly detection algorithms, to deal with more challenging malicious attack scenarios.

## 6. Conclusion

To address the multi-objective collaborative optimization challenges faced by UWSNs in complex dynamic environments, this study designs and implements a distributed intelligent power management scheme based on MARL. Its main methodological contributions lie in the following: Firstly, it has established a system simulation environment integrating high-fidelity channel models, passive node movement models, and random fault models, laying the foundation for the practicality of learning strategies. Secondly, a composite team reward function integrating fair reuse rewards and hard constraint penalties for lifetime is proposed, effectively guiding distributed agents to balance short-term throughput and long-term network survivability under partially observable conditions. Thirdly, a centralized training and distributed execution framework based on VDN is adopted, and a semi-cooperative learning mechanism is innovatively introduced, enabling the network to maintain robust performance even under irrational node interference. The simulation experiments verified the effectiveness and superiority of the proposed scheme. In a typical heterogeneous network scenario, this scheme achieves a total network capacity of 24,568.3 kb and a fair reuse index of 1.85 while ensuring that the network lifetime meets the task requirements. Compared with the advanced COMA, it has increased by 5.3% and 9.5%, respectively. When confronted with various non-ideal conditions such as random node failures, dynamic changes in communication thresholds, and water flow interference, this scheme demonstrates stronger adaptability and a more stable performance retention rate. For instance, it maintains a low average communication latency of 6.18 time slots at a 5% node failure rate.

## Supporting information

S1 File Minimal data set definition. (DOCX)
