# Supplementary material for: Power management and performance optimization of underwater wireless sensor networks based on MARL
Source: PLoS One. 2026 Mar 6;21(3):e0343529. doi: 10.1371/journal.pone.0343529 (PMC12965573; doi:10.1371/journal.pone.0343529)
Supplement: S1 File — (DOCX) [file pone.0343529.s001.docx]

The minimum dataset in Figure 7:

| Training round | Network multiplexing (number of links/time slots) | Fairness index | Network capacity (kb) | Network lifecycle (time slot) |
| --- | --- | --- | --- | --- |
| 1 | 1.2 | 0.50 | 5530 | 30 |
| 2.5×10^4^ | 2.8 | 0.75 | 12046 | 45 |
| 5.0×10^4^ | 3.5 | 0.85 | 18752 | 55 |
| 7.5×10^4^ | 4.0 | 0.90 | 22361 | 58 |
| 1.0×10^5^ | 4.3 | 0.93 | 24568 | 60 |

The minimum dataset in Table 2:

| NO. | 1 | 2 | 3 | 4 | 5 | 6 | 7 | 8 | 9 | 10 | Mean | Std |
| --- | --- | --- | --- | --- | --- | --- | --- | --- | --- | --- | --- | --- |
| FPA_Cap (kb) | 12562.1 | 12610.5 | 12545.8 | 12585.3 | 12602.4 | 12570.9 | 12595.7 | 12555.2 | 12588.6 | 12605 | 12580.6 | 23.7 |
| RPA_Cap (kb) | 15788.3 | 15845.2 | 15750.4 | 15820.1 | 15835.7 | 15795.6 | 15830.4 | 15765.8 | 15825.3 | 15840.9 | 15823.4 | 35.2 |
| APA_Cap (kb) | 18210.5 | 18275.8 | 18185.2 | 18250.4 | 18265.9 | 18220.8 | 18255.6 | 18195.7 | 18245.1 | 18270.5 | 18245.1 | 31.8 |
| RAPA_Cap (kb) | 16485.2 | 16530.1 | 16420.7 | 16495.8 | 16515.3 | 16465.4 | 16505.9 | 16435.6 | 16488.5 | 16525.4 | 16512.8 | 38.5 |
| COMA_Cap (kb) | 22015.4 | 22085.7 | 21975.6 | 22050.2 | 22070.8 | 22025.9 | 22060.5 | 21990.3 | 22045.7 | 22080.1 | 22037.9 | 37.4 |
| DMPM_Cap (kb) | 24530.8 | 24595.4 | 24485.1 | 24560.9 | 24580.5 | 24540.2 | 24570.8 | 24500.4 | 24555.3 | 24590.7 | 24568.3 | 42.1 |
| FPA_FRI | 0.94 | 0.96 | 0.93 | 0.95 | 0.96 | 0.94 | 0.95 | 0.93 | 0.95 | 0.96 | 0.95 | 0.012 |
| RPA_FRI | 0.71 | 0.73 | 0.7 | 0.72 | 0.73 | 0.71 | 0.72 | 0.7 | 0.72 | 0.73 | 0.72 | 0.011 |
| APA_FRI | 0.57 | 0.59 | 0.56 | 0.58 | 0.59 | 0.57 | 0.58 | 0.56 | 0.58 | 0.59 | 0.58 | 0.011 |
| RAPA_FRI | 0.4 | 0.42 | 0.39 | 0.41 | 0.42 | 0.4 | 0.41 | 0.39 | 0.41 | 0.42 | 0.41 | 0.011 |
| COMA_FRI | 1.6 | 1.63 | 1.59 | 1.62 | 1.64 | 1.61 | 1.63 | 1.6 | 1.62 | 1.64 | 1.62 | 0.017 |
| DMPM_FRI | 1.83 | 1.86 | 1.82 | 1.85 | 1.87 | 1.84 | 1.86 | 1.83 | 1.85 | 1.87 | 1.85 | 0.018 |
| FPA_Life | 44 | 46 | 45 | 45 | 46 | 44 | 45 | 44 | 45 | 46 | 45 | 0.9 |
| RPA_Life | 54 | 56 | 55 | 55 | 56 | 54 | 55 | 54 | 55 | 56 | 55 | 0.9 |
| APA_Life | 60 | 60 | 60 | 60 | 60 | 60 | 60 | 60 | 60 | 60 | 60 | 0 |
| RAPA_Life | 57 | 59 | 58 | 58 | 59 | 57 | 58 | 57 | 58 | 59 | 58 | 0.9 |
| COMA_Life | 60 | 60 | 60 | 60 | 60 | 60 | 60 | 60 | 60 | 60 | 60 | 0 |
| DMPM_Life | 60 | 60 | 60 | 60 | 60 | 60 | 60 | 60 | 60 | 60 | 60 | 0 |
| FPA_Delay | 3.9 | 3.7 | 4 | 3.8 | 3.6 | 3.9 | 3.7 | 4 | 3.8 | 3.6 | 3.8 | 0.15 |
| RPA_Delay | 2.6 | 2.4 | 2.7 | 2.5 | 2.3 | 2.6 | 2.4 | 2.7 | 2.5 | 2.3 | 2.5 | 0.15 |
| APA_Delay | 4.3 | 4.1 | 4.4 | 4.2 | 4 | 4.3 | 4.1 | 4.4 | 4.2 | 4 | 4.2 | 0.15 |
| RAPA_Delay | 3.6 | 3.4 | 3.7 | 3.5 | 3.3 | 3.6 | 3.4 | 3.7 | 3.5 | 3.3 | 3.5 | 0.15 |
| COMA_Delay | 1.6 | 1.4 | 1.7 | 1.5 | 1.3 | 1.6 | 1.4 | 1.7 | 1.5 | 1.3 | 1.5 | 0.13 |
| DMPM_Delay | 1.3 | 1.1 | 1.4 | 1.2 | 1 | 1.3 | 1.1 | 1.4 | 1.2 | 1 | 1.2 | 0.13 |

The minimum dataset in Figure 8:

| Training round | This Study | | COMA | |
| --- | --- | --- | --- | --- |
|  | Average Reward (D=3) | Average Reward (D=6) | Average Reward (D=3) | Average Reward (D=6) |
| 1 | 10.5 | 5.3 | 9.4 | 4.3 |
| 2.5×10^4^ | 45.2 | 30.1 | 44.6 | 17.1 |
| 5.0×10^4^ | 70.8 | 65.4 | 69.5 | 22.9 |
| 7.5×10^4^ | 88.3 | 85.9 | 77.2 | 23.4 |
| 1.0×10^5^ | 94.7 | 97.6 | 83.2 | 23.6 |

The minimum dataset in Figure 9:

| Training round | This Study | | COMA | |
| --- | --- | --- | --- | --- |
|  | Average Reward (D=3) | Average Reward (D=6) | Average Reward (D=3) | Average Reward (D=6) |
| 1 | 12.3 | 8.7 | 10.5 | 5.2 |
| 2.5×10^4^ | 52.8 | 48.1 | 45.2 | 18.6 |
| 5.0×10^4^ | 82.4 | 78.5 | 70.3 | 21.4 |
| 7.5×10^4^ | 94.1 | 90.2 | 79.8 | 22.1 |
| 1.0×10^5^ | 98.5 | 96.3 | 82.4 | 22.7 |

The minimum dataset in Table 3:

| NO. | 1 | 2 | 3 | 4 | 5 | Mean |
| --- | --- | --- | --- | --- | --- | --- |
| D2_FPA | 3.5 | 3.6 | 3.4 | 3.5 | 3.6 | 3.52 |
| D2_APA | 2 | 2.1 | 1.9 | 2 | 2.1 | 2.01 |
| D2_RPA | 4.8 | 4.9 | 4.7 | 4.8 | 5 | 4.83 |
| D2_COMA | 2.9 | 2.8 | 2.7 | 2.9 | 3 | 2.85 |
| D2_DMPM | 1.5 | 1.6 | 1.4 | 1.5 | 1.6 | 1.52 |
| D3_FPA | 12.8 | 12.9 | 12.7 | 12.7 | 13 | 12.71 |
| D3_APA | 3 | 3.1 | 2.9 | 3 | 3.2 | 3.02 |
| D3_RPA | 7.3 | 7.2 | 7.1 | 7.2 | 7.4 | 7.24 |
| D3_COMA | 4.1 | 4.2 | 4 | 4.1 | 4.3 | 4.13 |
| D3_DMPM | 2.3 | 2.4 | 2.2 | 2.3 | 2.5 | 2.31 |
| D4_FPA | 28.6 | 28.8 | 28.4 | 28.5 | 29 | 28.53 |
| D4_APA | 4 | 4.1 | 3.9 | 4 | 4.2 | 4.03 |
| D4_RPA | 10.9 | 11 | 10.8 | 10.9 | 11.1 | 10.87 |
| D4_COMA | 5.5 | 5.6 | 5.4 | 5.5 | 5.7 | 5.52 |
| D4_DMPM | 3.7 | 3.6 | 3.5 | 3.6 | 3.8 | 3.64 |
| D5_FPA | 55.2 | 55 | 54.8 | 55.1 | 55.3 | 55.14 |
| D5_APA | 5 | 5.1 | 4.9 | 5 | 5.2 | 5.01 |
| D5_RPA | 15.4 | 15.3 | 15.2 | 15.3 | 15.5 | 15.31 |
| D5_COMA | 7.4 | 7.3 | 7.2 | 7.3 | 7.5 | 7.38 |
| D5_DMPM | 4.8 | 4.9 | 4.7 | 4.8 | 5 | 4.82 |
| D6_FPA | 60 | 60 | 60 | 60 | 60 | 60 |
| D6_APA | 6 | 6.1 | 5.9 | 6 | 6.2 | 6.02 |
| D6_RPA | 21.7 | 21.6 | 21.5 | 21.6 | 21.8 | 21.65 |
| D6_COMA | 9.8 | 9.9 | 9.7 | 9.8 | 10 | 9.76 |
| D6_DMPM | 6.2 | 6.1 | 6 | 6.2 | 6.3 | 6.18 |

The minimum dataset in Table 4:

| NO. | 1 | 2 | 3 | 4 | 5 | Mean |
| --- | --- | --- | --- | --- | --- | --- |
| FPA_Cap (kb) | 8310.2 | 8325.7 | 8315.4 | 8330.1 | 8325.1 | 8321.5 |
| COMA_Cap (kb) | 17045.8 | 17065.4 | 17035.1 | 17075.9 | 17068.8 | 17056.2 |
| DMPM_Cap (kb) | 19418.5 | 19438.9 | 19408.2 | 19448.5 | 19435.4 | 19428.7 |
| FPA_Delay | 17.9 | 17.7 | 18 | 17.6 | 17.8 | 17.8 |
| COMA_Delay | 8.6 | 8.4 | 8.7 | 8.3 | 8.5 | 8.5 |
| DMPM_Delay | 5.5 | 5.3 | 5.6 | 5.2 | 5.4 | 5.4 |
| FPA_PDR (%) | 68.8 | 69.3 | 68.5 | 69.5 | 69.2 | 69.1 |
| COMA_PDR (%) | 87 | 87.5 | 86.8 | 87.7 | 87.3 | 87.3 |
| DMPM_PDR (%) | 91.8 | 92.3 | 91.5 | 92.5 | 92.1 | 92.1 |
| FPA_EE (kb/J) | 0.221 | 0.222 | 0.22 | 0.223 | 0.222 | 0.222 |
| COMA_EE (kb/J) | 0.339 | 0.341 | 0.338 | 0.342 | 0.34 | 0.34 |
| DMPM_EE (kb/J) | 0.388 | 0.391 | 0.387 | 0.392 | 0.389 | 0.389 |

The minimum dataset in Figure 10:

| SINR Threshold (dB) | FPA (kb) | APA (kb) | COMA (kb) | RPA (kb) | RAPA (kb) | This Study (kb) |
| --- | --- | --- | --- | --- | --- | --- |
| HomNet | | | | | | |
| 10 | 7820.5 | 12450.2 | 15840.8 | 11580.3 | 9805.7 | 32005.4 |
| 12 | 6502.1 | 11875.6 | 15210.5 | 10425.8 | 8920.1 | 31968.9 |
| 14 | 5210.8 | 10985.3 | 14563.2 | 9220.7 | 8015.4 | 31920.5 |
| 16 | 4125.3 | 9950.7 | 13875.9 | 8240.2 | 7210.8 | 31875.1 |
| 18 | 3150.6 | 9015.4 | 13205.6 | 7315.6 | 6502.3 | 31810.2 |
| HetNet | | | | | | |
| 10 | 4980.2 | 7825.4 | 12050.3 | 6820.5 | 5985.1 | 24805.7 |
| 12 | 4210.5 | 7320.8 | 11520.6 | 6102.8 | 5420.3 | 24760.4 |
| 14 | 3515.8 | 6815.2 | 11010.9 | 5520.1 | 4925.6 | 24715.2 |
| 16 | 2850.3 | 6320.5 | 10520.4 | 4980.7 | 4480.9 | 24670.8 |
| 18 | 2245.6 | 5825.1 | 10015.7 | 4510.4 | 4050.2 | 24625.3 |

The minimum dataset in Figure 11:

| Water Flow Velocity (m/s) | FPA (kb) | RPA (kb) | APA (kb) | COMA (kb) | This Study (kb) |
| --- | --- | --- | --- | --- | --- |
| HomNet | | | | | |
| 0.4 | 21850.3 | 23500.7 | 27520.5 | 33800.9 | 40250.6 |
| 0.8 | 20500.8 | 22800.2 | 27210.3 | 33250.4 | 39980.2 |
| 1.2 | 19250.1 | 22050.6 | 26850.8 | 32800.7 | 39720.5 |
| 1.6 | 17800.5 | 21200.9 | 26400.4 | 32350.1 | 39450.3 |
| 2 | 16500.2 | 20500.3 | 25950.7 | 31900.6 | 39180.9 |
| HetNet | | | | | |
| 0.4 | 19500.5 | 21500.8 | 24800.2 | 31500.6 | 38800.4 |
| 0.8 | 18250.7 | 20800.3 | 24350.7 | 31000.9 | 38520.1 |
| 1.2 | 17000.2 | 20050.5 | 23800.4 | 30500.3 | 38250.8 |
| 1.6 | 15800.6 | 19200.9 | 23350.6 | 30000.7 | 37980.5 |
| 2 | 14500.3 | 18500.2 | 22800.3 | 29500.2 | 37650.1 |

The minimum dataset in Figure 12:

| Network Capacity | | | | | |
| --- | --- | --- | --- | --- | --- |
| Mobile Node Power (W) | Greedy (kb) | Random (kb) | TDMA (kb) | CQL (kb) | This Study (kb) |
| 4 | 24850.3 | 18200.7 | 12500.5 | 9500.2 | 34280.6 |
| 8 | 19500.8 | 15000.3 | 11000.1 | 8200.5 | 31500.4 |
| 16 | 14200.5 | 11500.9 | 9500.7 | 6800.3 | 28500.2 |
| 32 | 8500.2 | 7500.4 | 7000.8 | 4800.6 | 23800.7 |
| 64 | 4500.6 | 4000.1 | 4500.3 | 2500.9 | 20500.3 |
| Concurrency Rate | | | | | |
| Mobile Node Power (W) | Greedy | Random | TDMA | CQL | This Study |
| 4 | 0.32 | 0.25 | 0.18 | 0.12 | 0.52 |
| 8 | 0.28 | 0.22 | 0.16 | 0.1 | 0.48 |
| 16 | 0.23 | 0.18 | 0.14 | 0.08 | 0.42 |
| 32 | 0.15 | 0.12 | 0.1 | 0.05 | 0.35 |
| 64 | 0.08 | 0.07 | 0.06 | 0.03 | 0.28 |
| Energy Efficiency | | | | | |
| Mobile Node Power (W) | Greedy (kb/J) | Random (kb/J) | TDMA (kb/J) | CQL (kb/J) | This Study (kb/J) |
| 4 | 0.25 | 0.2 | 0.15 | 0.11 | 0.83 |
| 8 | 0.22 | 0.18 | 0.13 | 0.09 | 0.72 |
| 16 | 0.18 | 0.15 | 0.11 | 0.07 | 0.6 |
| 32 | 0.12 | 0.1 | 0.08 | 0.04 | 0.48 |
| 64 | 0.06 | 0.05 | 0.05 | 0.02 | 0.39 |

The minimum dataset in Table 5:

| NO. | STM32_Time (ms) | STM32_Mem (KB) | STM32_Energy (mJ) | Arduino_Time (ms) | Arduino_Mem (KB) | Arduino_Energy (mJ) |
| --- | --- | --- | --- | --- | --- | --- |
| 1 | 37.8 | 18.4 | 1.88 | 101.5 | 19.2 | 5.05 |
| 2 | 38.5 | 18.6 | 1.92 | 103.2 | 19.4 | 5.12 |
| 3 | 37.5 | 18.3 | 1.85 | 102 | 19.1 | 5.08 |
| 4 | 38 | 18.5 | 1.9 | 102.8 | 19.3 | 5.1 |
| 5 | 38.2 | 18.6 | 1.91 | 103.5 | 19.5 | 5.15 |
| 6 | 37.9 | 18.4 | 1.89 | 101.7 | 19.2 | 5.06 |
| 7 | 38.3 | 18.5 | 1.93 | 102.3 | 19.3 | 5.09 |
| 8 | 37.6 | 18.3 | 1.86 | 102.5 | 19.4 | 5.11 |
| 9 | 38.1 | 18.5 | 1.9 | 103 | 19.3 | 5.13 |
| 10 | 38.4 | 18.6 | 1.94 | 102.2 | 19.2 | 5.07 |
| Mean | 38.2 | 18.5 | 1.9 | 102.7 | 19.3 | 5.1 |
| Std | 0.35 | 0.12 | 0.032 | 0.68 | 0.12 | 0.036 |

The minimum dataset in Table 6:

| NO. | 1 | 2 | 3 | 4 | 5 | 6 | 7 | 8 | 9 | 10 | Mean | Std |
| --- | --- | --- | --- | --- | --- | --- | --- | --- | --- | --- | --- | --- |
| DMPM_Cap (KB) | 25480.5 | 25400.8 | 25520.1 | 25360.4 | 25490.6 | 25410.9 | 25530.2 | 25370.5 | 25480.7 | 25421 | 25432.7 | 415.3 |
| MADDPG_Cap (KB) | 19910.2 | 19820.5 | 19980.8 | 19740.1 | 19930.3 | 19840.6 | 20000.9 | 19750.2 | 19920.4 | 19850.7 | 19875.4 | 632.8 |
| VDN_Cap (KB) | 21085.4 | 20975.1 | 21145.7 | 20885 | 21095.5 | 20985.8 | 21155.9 | 20895.2 | 21085.6 | 20995.9 | 21023.6 | 587.1 |
| QMIX_Cap (KB) | 24160.8 | 24050.3 | 24220.9 | 23940.2 | 24170.4 | 24060.7 | 24230.1 | 23950.5 | 24160.6 | 24070.8 | 24105.9 | 498.6 |
| COMA_Cap (KB) | 22600.7 | 22490.2 | 22700.8 | 22380.1 | 22610.5 | 22500.8 | 22720.9 | 22390.3 | 22600.6 | 22520.9 | 22547.3 | 560.2 |
| DMPM_FRI | 1.92 | 1.9 | 1.93 | 1.89 | 1.92 | 1.9 | 1.93 | 1.89 | 1.92 | 1.91 | 1.91 | 0.07 |
| MADDPG_FRI | 1.36 | 1.34 | 1.37 | 1.33 | 1.36 | 1.34 | 1.38 | 1.33 | 1.36 | 1.35 | 1.35 | 0.13 |
| VDN_FRI | 1.43 | 1.41 | 1.44 | 1.4 | 1.43 | 1.41 | 1.45 | 1.4 | 1.43 | 1.42 | 1.42 | 0.11 |
| QMIX_FRI | 1.81 | 1.79 | 1.82 | 1.78 | 1.81 | 1.79 | 1.83 | 1.78 | 1.81 | 1.8 | 1.8 | 0.09 |
| COMA_FRI | 1.69 | 1.67 | 1.7 | 1.66 | 1.69 | 1.67 | 1.71 | 1.66 | 1.69 | 1.68 | 1.68 | 0.12 |
| DMPM_Delay | 1.1 | 1.2 | 1 | 1.3 | 1.15 | 1.25 | 0.95 | 1.35 | 1.12 | 1.22 | 1.15 | 0.18 |
| MADDPG_Delay | 2.1 | 2.2 | 1.9 | 2.3 | 2.15 | 2.25 | 1.85 | 2.35 | 2.12 | 2.22 | 2.05 | 0.31 |
| VDN_Delay | 1.9 | 2 | 1.8 | 2.1 | 1.95 | 2.05 | 1.75 | 2.15 | 1.92 | 2.02 | 1.88 | 0.28 |
| QMIX_Delay | 1.4 | 1.5 | 1.3 | 1.6 | 1.45 | 1.55 | 1.25 | 1.65 | 1.42 | 1.52 | 1.38 | 0.21 |
| COMA_Delay | 1.55 | 1.65 | 1.45 | 1.75 | 1.6 | 1.7 | 1.4 | 1.8 | 1.57 | 1.67 | 1.52 | 0.24 |
